# GCS overexpression is associated with multidrug resistance of human HCT-8 colon cancer cells

**DOI:** 10.1186/1756-9966-31-23

**Published:** 2012-03-16

**Authors:** Min Song, Weidong Zang, Baohua Zhang, Jing Cao, Guanrui Yang

**Affiliations:** 1 Department of Oncology, The First Affiliated Hospital of Zhengzhou University, Zhengzhou 450052, Henan, P.R. China; 2 Department of Human Anatomy, College of Basic Medical Science, Zhengzhou University, Zhengzhou 450001, Henan, P.R. China; 3 Henan Academy of Medical and Pharmaceutical Sciences, Zhengzhou University, 40 Daxue Road, Zhengzhou 450052, Henan, P.R. China

**Keywords:** Glucosylceramide synthase, RNA interference, Multidrug resistance, P-gp

## Abstract

**Purpose:**

Multidrug resistance is one of the main impediments to the successful treatment of colon cancer. Glucosylceramide synthase (GCS) which is related to multidrug resistance (MDR) can reduce the level of ceramide and can help cells escape from the ceramide-induced cell apoptosis. However, the underlying mechanism is still unclear.

**Methods:**

The cell proliferation and cell toxicity were measured with Cell Counting Kit-8 (CCK-8). The mRNA levels of GCS and MDR1 were detected by semiquantitative reverse transcription-PCR amplification, the protein levels of GCS, caspase-3 and P-gp proteins were indicated by Western blotting. The apoptosis rates of cells were measured with flow cytometry.

**Results:**

The relative mRNA levels of GCS in HCT-8, HCT-8/VCR, HCT-8/VCR- sh-mock and HCT-8/VCR-sh-GCS were 71.4 ± 1.1%, 95.1 ± 1.2%, 98.2 ± 1.5%, and 66.6 ± 2.1% respectively. The mRNA levels of MDR1 were respectively 61.3 ± 1.1%, 90.5 ± 1.4%, 97.6 ± 2.2% and 56.1 ± 1.2%. The IC50 of Cisplatin complexes were respectively 69.070 ± 0.253 μg/ml, 312.050 ± 1.46 μg/ml, 328.741 ± 5.648 μg/ml, 150.792 ± 0.967 μg/ml in HCT-8, HCT-8/VCR, HCT-8/VCR-sh-mock and HCT-8/VCR-sh-GCS. The protein levels of caspase-3 were 34.2 ± o.6%, 93.0 ± 0.7%, 109.09 ± 0.7%, 42.7 ± 1.3% respectively. The apoptosis rates of cells were 8.77 ± 0.14%, 12.75 ± 0.54%, 15.39 ± 0.41% and 8.49 ± 0.23% respectively.

**Conclusion:**

In conclusion, our research indicated that suppression of GCS restores the sensitivity of multidrug resistance colon cancer cells to drug treatment.

## Background

Multidrug resistance (MDR) is one of the main impediments to the successful treatment of colon cancer [1]. Furthermore, colorectal tumors which obtain resistance to one drug are often resistant to several other drugs as well [2]. The underlying mechanisms are complicated [3]. One reason for MDR relates to P-glycoprotein (P-gp) and other transporters which are expressed in some cancer cells and could strengthen the efflux of diverse chemotherapeutic agents from cells [2]. Elevated levels of these MDR proteins, which belong to the ATP-binding cassette (ABC) transporter family, strengthen cellular efflux and reduce the effectiveness of anticancer drugs [4]. One method to measure P-glycoprotein efflux has been set up to o determine tumor response to chemotherapy [1]. To conquer drug resistance, inhibitors of MDR proteins have been developed, however their non-specific inhibition has brought side effects.

Glucosylceramide (GCS) can reduce the level of ceramide and allows cellular escape from ceramide-induced cell apoptosis, which has been deemed to be related with MDR [5]. More recently, it has been demonstrated that the expression of the GCS gene in drug-resistant K562/AO2 human leukemia cells was higher than that in drug-sensitive K562 cells, and the sensitivity of K562/AO2 cells to adriamycin was enhanced by GCS inhibition [6].

The mechanisms mediating drug resistance include defective apoptotic signaling and overexpression of anti-apoptotic proteins, which regulate apoptotic cell death and which also play an important role in determining the sensitivity of tumor cells to chemotherapy [7]. High level expression of Bcl-2 is found in many human hematologic malignancies and solid tumors [8,9]. The downregulation of Bcl-2 or other anti-apoptotic proteins, such as Bcl-xL, could either induce apoptosis in cancer cells or could sensitize these cells for chemotherapy [10,11]. In addition, these proteins protect drug-resistant tumor cells from multiple forms of caspase-dependent apoptosis [12,13]. Moreover, functional P-gp can inhibit the activation of caspase-3 and-8 by some apoptotic stimuli [14,15].

Based on the above, we speculate that suppression of GCS by the stable transfection of UGCG shRNA Plasmid would restore sensitivity of multidrug resistance colon cancer cells by the stable transfection of UGCG shRNA Plasmid.

## Methods

### Cell lines and cell culture

The colon cancer cell line HCT-8 was purchased from ATCC, and the cell line HCT-8/VCR was purchased from Xiangya Central Experiment Laboratory (Hunan, China). The cells were cultured at 37°C in RPMI-1640 culture medium (Hyclone) in humidified atmosphere containing 5% CO_2_, with the medium for HCT-8 cells containing 10% FBS, and with the medium for HCT-8/VCR cells containing 10% FBS and 2 μg/ml vincristine. All experiments were performed according to the guidelines approval by The ethical committee of Zhengzhou University(NO.20120066).

### Stable transfection of cells

UGCG shRNA Plasmid (h) was purchased from Santa Cruz. UGCG shRNA Plasmid (h) is recommended for the inhibition of glucosylceramide synthase expression in human cells, which is a pool of 3 target-specific lentiviral vector plasmids encoding 19-25 nt (plus hairpin) shRNAs designed to knock down gene expression. HCT-8 cells were seeded in 6-well plate with antibiotic free medium. After 24 h incubation, the mixture of transfection regent and ShRNA were incubated with cells according to the manufacturer's instructions. These cells were incubated for an additional 18-24 hours under normal culture conditions. 48 h after transfection, the medium was aspirated and replaced with fresh medium containing 100 μg/ml puromycin. The medium was changed every 3 days. The following experiments were performed after 20 days of culture.

### Semi- quantitative reverse transcription-PCR analysis

Total RNA was extracted from cells by using TRIquick Reagent (solarbio). The mRNA levels of GCS and MDR1 were measured with RT-PCR. The following specific oligonucleotide primers were designed respectively for mdr1 (mdr1-F:5'- TGGTGGTGGGAACTT TGG-3' and mdr1-R:5'-CCTATCTCCTGTCGCATT-3'), GCS (GCS-F:5'-CACCCGATTACACCTCAA - 3' and GCS-R: 5'-CCGTGAACC AAGCCTACT-3'), β-actin (β-actin-F:5'-TGACGTGGACATC CGCAAAG - 3', and β-actin-R: 5'-CTGGAA GGTGGACAGCGAGG - 3'). PCR cycles were adjusted to have linear amplification for all the targets. Each RT-PCR reaction was repeated three times. The semiquantitative analysis of GCS mRNA and MDR1 mRNA levels was measured with Syngene Gel Imaging System and analysis software (Syngene Company).

### Western blotting analysis of P-gp, Caspase-3 and GCS protein

HCT-8, HCT-8/VCR, HCT-8/VCR-sh-mock and HCT-8/VCR-sh-GCS were harvested using RIPA cell lysis buffer (Biotech Corporation). The protein concentrations were measured by using a bicinchoninic acid (BCA) protein assay kit. Equal aliquots of total detergent-soluble proteins (50 μg) were resolved to 5-10% gradient SDS-PAGE. The transferred PVDF membrane were blocked with 5% fat-free milk in TBST at room temperature for 1 h and then incubated with primary antibodies (anti-P-gp antibody, C-19, anti-GCS antibody, anti-caspase-3 or anti-β-actin antibody; 1:1000 dilution) at 4°C overnight. The protein was detected by using horseradish peroxidase (HRP) and enzyme-linked chemiluminescence (ECL) plus substrate (GE Healthcare, Piscataway, NJ) Anti-human P-gp antibody (C-19) and GCS antibody (H-300) and anti-caspase-3 antibody were purchased from Santa Cruz Corporation. The protein levels of P-gp, Caspase-3 and GCS were represented by the ratios of optical densities in their bands normalized against β-actin.

### Cytotoxicity assay

In 96 well plates, cells were seeded in 100 μl PRMI-1640 medium supplemented with 10% FBS at 5 × 10^3 ^cells/well. Then chemotherapeutic agents were added in normal growth medium supplemented with FBS. After 48 h incubation, 10 μl Cell Counting Kit-8 (CCK-8) was added and culture was continued for 1 h in humidified atmosphere containing 5% CO_2_. Absorbances at 450 nm were measured by Microplate Reader (Bio-Tech Company). The relative drug resistance folds were analyzed by compared with IC50.

### Flow cytometry

To measure the apoptosis rate of the cells, we chose the AnnexinV-FITC Apoptosis Detection Kit. The cells were washed 2 times by 4°CPBS, and diluted with 400 μl AnnexinV binding liquid, then added 5 μl Annexin V-FITC at 4°C for 15 min without light, and then added 10 μl PI at 4°C for 5 min without light. The cells were measure with flow cytometry within 1 h.

### Statistical analysis

All of the data were presented as the mean ± SD, and analyzed with one-way ANOVA by SPSS16.0 software package.

## Results

### mRNA levels of GCS and MDR1 decreased in the cells stable transfected with UGCG shRNA Plasmid (h)

**Figure 1:**
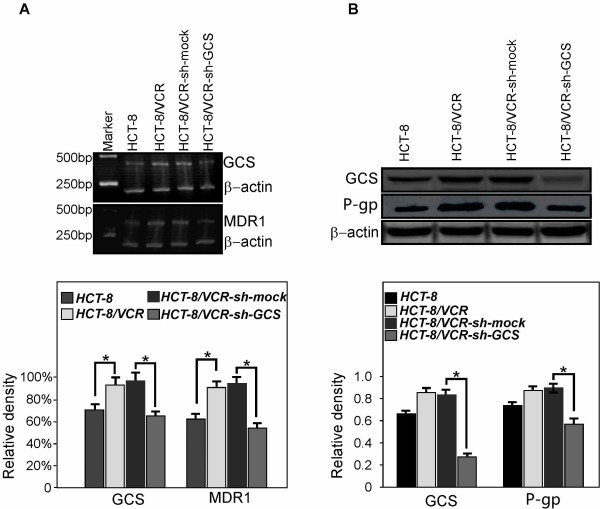
**Knocking down GCS inhibits mRNA expression of MDR1 and protein level of P-pg**. A, the mRNA level are higher in HCT-8/VCR cells compared with HCT-8 cells. The GCS mRNA level decreased when transfected with shGCS plasmids. The MDR1 gene expressin increased in HCT-8/VCR cells compared with HCT-8 cells. The MDR1 mRNA level also decreased when knocking down GCS. B, the protein level of P-pg decreased when knocking down GCS. Protein level of β-actin was set as 100%. *Ρ < 0.01 compared with the HCT-8/VCR and HCT-8/VCR-sh-mock cells.

To check the knockdown effect of UGCG shRNA, the mRNA levels of GCS and MDR1 were measured with semiquantitative reverse transcription-PCR amplification. The length of the PCR products were 331 bp (MDR1), 414 bp (GCS) and 205 bp (β-actin) respectively. As shown in Figure 1A, the relative mRNA levels of GCS in HCT-8, HCT-8/VCR, HCT-8/VCR-sh-mock and HCT-8/VCR-sh-GCS were 71.4 ± 1.1%, 95.1 ± 1.2%, 98.2 ± 1.5%, and 66.6 ± 2.1% respectively. The mRNA levels of MDR1 were respectively 61.3 ± 1.1%, 90.5 ± 1.4%, 97.6.8 ± 2.2% and 56.1 ± 1.2%.

### P-gp protein level decreased when knocking down GCS in HCT-8/VCR cells

The protein levels of GCS and P-gp in stable cell lines were detected by Western-blotting. As indicated in Figure 1B, the protein level of GCS increased in HCT-8/VCR, HCT-8/VCR-sh-mock cells compared to HCT-8 cells. The protein levels of GCS in HCT-8/VCR-sh-GCS decreased when transfected with Sh-GCS(*Ρ < 0.01*). It also true for protein level of P-pg.

### Knocking down GCS suppressed HCT-8/VCR proliferation

**Figure 2:**
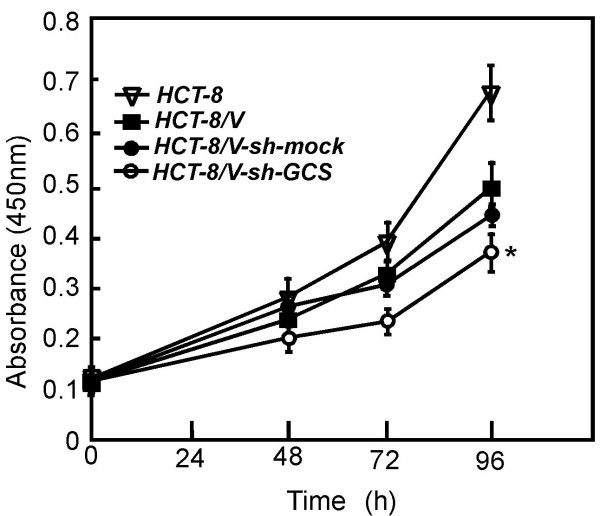
**Knocking down GCS suppresses HCT-8/VCR cell proliferation**. HCT-8 cell (2 × 10^3^) were seeded in 96-well in 100 ul PRMI-1640 medium. Cell proliferation was determined at 24-h intervals up to 96 h in sh-mock or sh-GCS stably transfected cells. Data are shown as means ± S.D.

The proliferation of HCT-8, HCT-8/VCR, HCT-8/VCR-sh-mock and HCT-8/VCR-sh-GCS cells was detected by Cell Counting Kit-8 (CCK-8). We measured the growth of the cells every 24 h, for 4 days. Knowing down GCS impaired HCT-8/VCR-sh-GCS cell proliferation (*Ρ *< 0.05) (Figure 2).

### Knocking down GCS in HCT-8/VCR cells reverse its sensitive to cisplatin treatment

**Figure 3:**
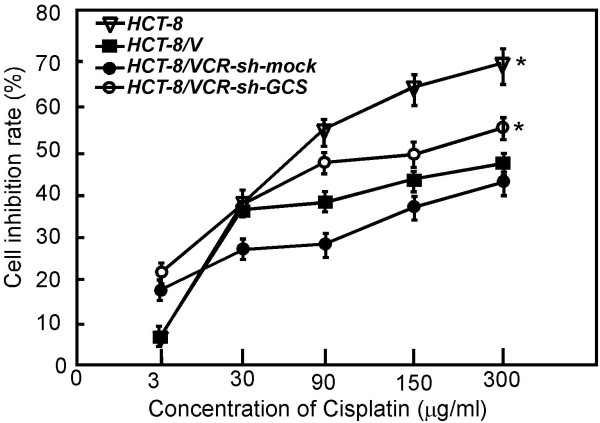
**Knocking down GCS causes HCT-8/VCR more sensitive to cisplatin induced cell death**. HCT-8, HCT-8/VCR, HCT-8/VCR sh-mock or sh-GCS stably transfected cells (5 × 10^3^) were seeded in 96-well in 100 ul PRMI-1640 medium. Cells were treated with different concentaration of cisplatin for 48 h. The relative drug resistance folds were analyzed by compared with IC50. The cell survival rate of cells without cisplatin complexes was set as 100%. *Ρ < 0.01 compared with the HCT-8/VCR and HCT-8/VCR-sh-mock cells.

Cisplatin is one of the effective chemotherapeutic agents in clinical cancer treatment. It was found here that the IC50 of Cis-platinum complexes were respectively 69.070 ± 0.253 μg/ml, 312.050 ± 1.46 μg/ml, 328.741 ± 5.648 μg/ml, 150.792 ± 0.967 μg/ml in HCT-8, HCT-8/VCR, HCT-8/VCR-sh-mock and HCT-8/VCR-sh-GCS. The drug resistance folds were respectively 4.6 (HCT-8/VCR), 4.7(HCT-8/VCR-sh-mock), 2.2(HCT-8/VCR-sh-GCS), the sensitive cells HCT-8 was set as 1(Figure 3).

### Knocking down GCS positively related with caspase-3 protein level in HCT-8/VCR cells

**Figure 4:**
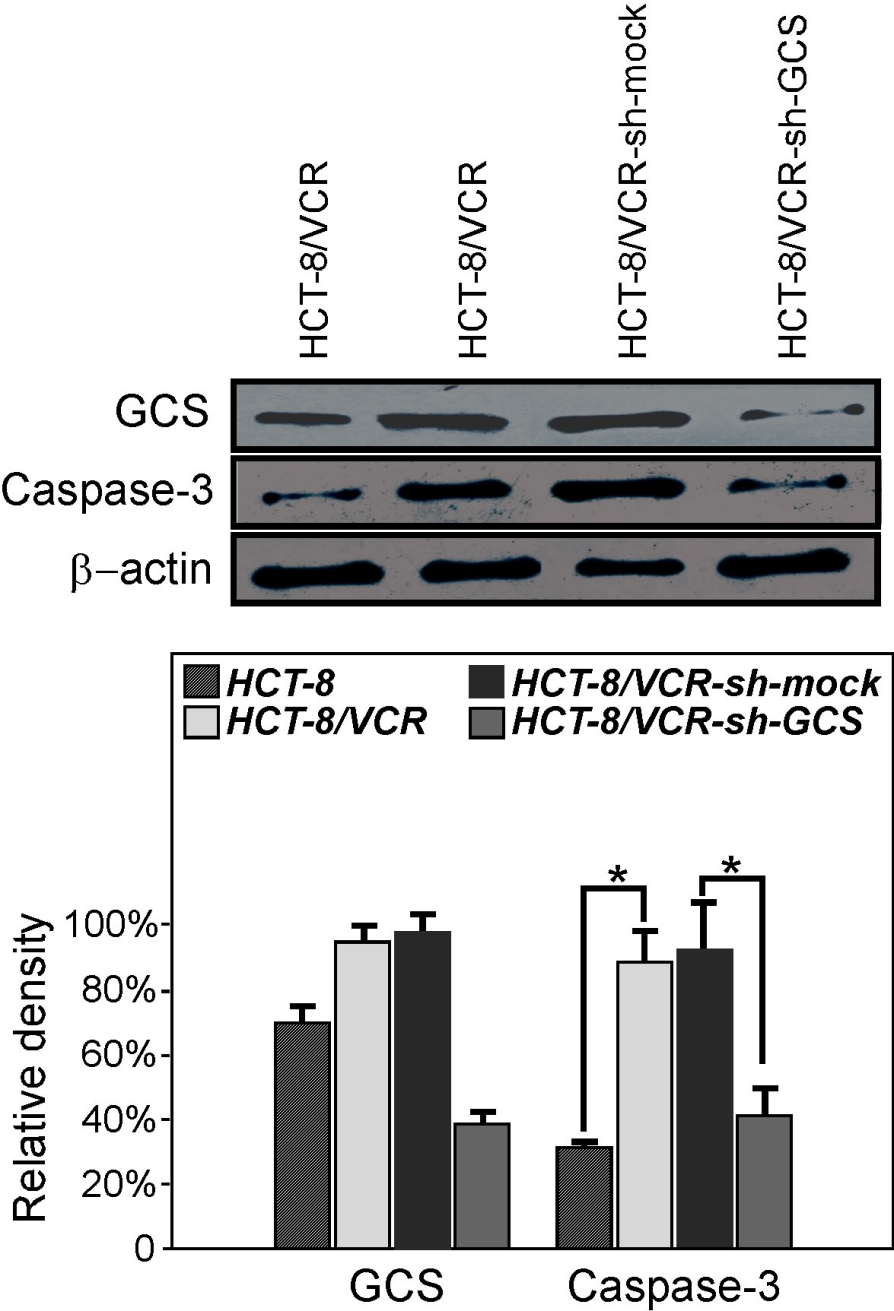
**Knocking down GCS affects Caspase-3 protein level**. The Caspase-3 protein level decreased when transfected with shGCS plasmids.

The downregulation of Bcl-2 or other antiapoptotic proteins could either induce apoptosis in cancer cells or sensitize these cells to chemotherapy [10,11]. Moreover, functional P-gp inhibits the activation of caspase-3 by some apoptotic stimuli [14,15]. We measured the protein levels of caspase-3 in HCT-8, HCT-8/VCR, HCT-8/VCR-sh-mock and HCT-8/VCR-sh-GCS cells. As shown in Figure 4 the relative expression levels of caspase-3 were respectively 34.2 ± 0.6%, 93.0 ± 0.7%, 109.09 ± 0.7%, 42.7 ± 1.3%.

### HCT-8/VCR cells apoptosis decreased in GCS knockdown HCT-8/VCR cells

**Figure 5:**
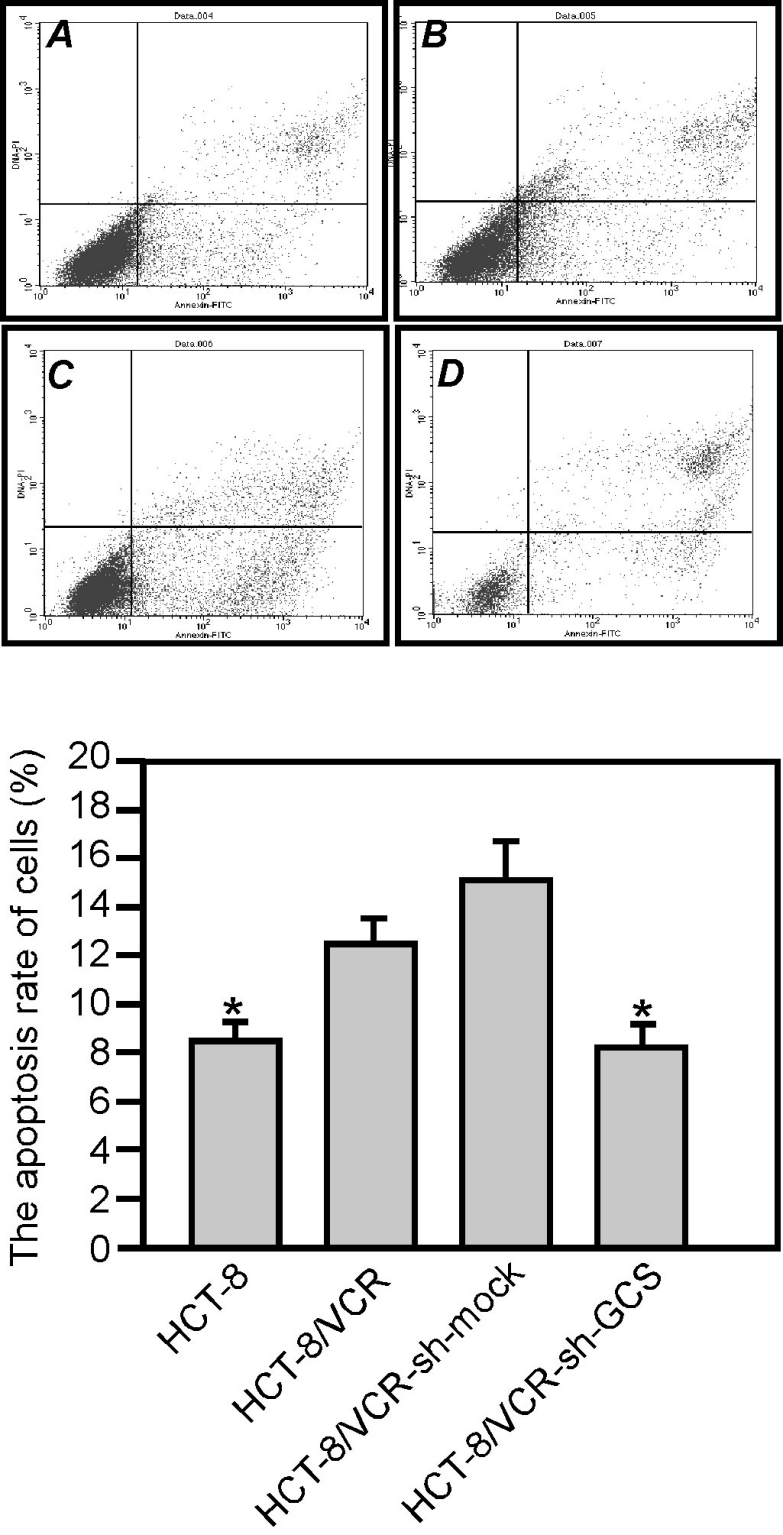
**Knocking down GCS affects HCT-8/VCR cells apoptosis**. The apoptosis of HCT-8, HCT-8/VCR, HCT-8/VCR sh-mock or sh-GCS stably transfected cells were measured with flow cytometry (A, HCT-8, B, HCT-8/VCR, C, HCT-8/VCR-sh-mock and D, HCT-8/VCR-sh-GCS).

The mechanisms mediating drug resistance include defective apoptotic signaling that regulate apoptotic cell death playing an important role in determining the sensitivity of tumor cells to chemotherapy [7]. We measured the apoptosis rates of cells by flow cytometry. The rates were shown in Figure 5, it demonstrated that the rates were 8.77 ± 0.14%, 12.75 ± 0.54%, 15.39 ± 0.41% and 8.49 ± 0.23%. By further analysis, there were differences in HCT-8, and HCT-8/VCR compared to HCT-8/VCR-sh-mock and HCT-8/VCR-sh-GCS(*Ρ *< 0.01). There were differences between HCT-8/VCR-sh -mock and HCT-8/VCR-sh-GCS (*Ρ *< 0.01).

## Discussion

Multidrug resistance is one of the main obstacles to the successful treatment in patients with colon cancer, and the underlying mechanisms are complex [1]. It is known that P-glycoprotein (P-gp), the drug efflux protein, and inhibitors of apoptosis proteins (IAPs) are involved in the MDR of leukemic cells [16]. Recently research has indicated that overexpression of P-gp and cIAP may enhance the infiltration of leukemic cells [16]. Lavie et al. revealed that chemotherapy resistant MCF-7-AdrR breast cancer cells accumulate GC compared to wild-type MCF-7 cells [17]. Furthermore, GCS has been found to confer MDR in many other cancers [18-20]. The level of protein P-gp in MCF-7-AdrR is higher than that in MCF-7. The GCS expression in these two cell lines has the same pattern. These phenomena give us the clue that these two proteins are closely related. The high expression of GCS in the same cell lines shows us that there may be some relation between P-gp and GCS.

Our results indicated that the mRNA level of GCS in HCT-8/VCR was higher than that in HCT-8, and its level decreased when the HCT-8/VCR were transfected with UGCG shRNA Plasmid. The expression level of MDR1 mRNA was also in the same pattern. This result indicated these two proteins have some relations. This result is consistent with the recently published work by liu et al. [21]. We also found that the protein level of caspase-3 was higher in insensitive cells than in sensitive cells.

Our research also found that the expression of GCS protein was much higher in HCT-8/VCR than that in HCT-8. And so was the protein level of P-gp. When the HCT-8/VCR was transfected with UGCG shRNA Plasmid, the protein levels of GCS and P-gp were decreased. The results indicated that there may be a relation between GCS and P-gp proteins.

Cytotoxity results demonstrated that HCT-8/VCR needs a much higher drug concentration to get 50% inhibition of cell growth. The needed drug concentration decreased when HCT-8/VCR was transfected with UGCG shRNA Plasmid. This result indicated that drug resistance in HCT-8/VCR was reversed. The higher level of the apoptotic gene in the insensitive cells may contribute to the result. Although the drugs can induce apoptosis, the cells with high level GCS may be better able to adapt to the new circumstances, while the sensitive cells may not. The apoptosis rate was higher in insensitive cells than sensitive cells. The result is different with the other researchers. The reason may be the coactions of many apoptotic and anti-apoptotic proteins.

In conclusion, our research demonstrated that GCS play an important role in multidrug resistance mechanisms of colon cancer cells with high expression of GCS gene. The up-regulation of GCS could affect the expression of MDR1 in colon cancer cells. They may cooperate with each other in the formation of multidrug resistance.

## Competing interests

The authors declare that they have no competing interests.

## Authors' contributions

MS and WD performed PCR, western blotting, and drafted the manuscript. BH performed total RNA preparation and reverse transcription. GR and JC conceived of the study and guided the biochemical experiments. All authors read and approved the final manuscript.
